# Late-life coronary heart disease mortality of Finnish war veterans in the TAMRISK study, a 28-year follow-up

**DOI:** 10.1186/1471-2458-11-71

**Published:** 2011-02-01

**Authors:** Tarja Kunnas, Tiina Solakivi, Jaana Renko, Anne Kalela, Seppo T Nikkari

**Affiliations:** 1 Department of Medical Biochemistry, University of Tampere Medical School, Tampere, Finland; 2 Tullinkulma Occupational Health Unit, City of Tampere, Tampere, Finland

## Abstract

**Background:**

Wartime stress has been associated with increased late-life mortality of all causes of death. We evaluated whether wounded Finnish World War II veterans who were alive at the age of 55 have increased long-term coronary heart disease (CHD) mortality.

**Methods:**

Health survey data were recorded in 1980 from 667 men, aged 55 years. Of them 102 had been wounded or injured in action during 1939-1945. The remaining participants served as the comparison group. The death certificates during a 28-year follow-up were obtained from the national statistics centre. Statistical comparisons were done by Cox proportional hazard regression model.

**Results:**

There were altogether 140 deaths from CHD. In men who had been wounded or injured in action the crude CHD mortality rate per 10,000 population was 2843, while in the comparison group the corresponding figure was 1961. Men who had been wounded or injured in action were 1.7 times (95% CI 1.1-2.5; p = 0.01) more likely to die from CHD than the comparison group.

**Conclusions:**

Physical trauma at young adulthood may extend to lifelong effects on health. This study suggests that being physically wounded or injured in war may lead to increased CHD mortality in late adulthood in a Finnish population.

## Background

There exist a multitude of studies that have investigated the impact of combat exposure on long-term health. A study from the Netherlands has provided evidence for increased late-life mortality among World War II military veterans approximately six decades later. Particularly wounded war survivors and those with posttraumatic stress disorder (PTSD) were at risk for death by any cause [1]. Vietnam veterans had statistically significant excesses of deaths from cancer and external causes, including motor vehicle accidents and accidental poisonings, in a 24-year retrospective analysis [2]. Among veterans of the Persian Gulf War, there was a significantly higher mortality rate than among veterans deployed elsewhere, but most of the increase was due to motor vehicle accidents rather than disease [3], an effect which dissipated after seven years of follow-up [4]. The same pattern has been observed also in UK Gulf War veterans [5]. The death rates of Australian Vietnam veterans were higher than those for other veterans for diseases of the digestive system, possibly indicating increased incidence of stress-related disorders [6].

There are only a few studies addressing the long-term cardiovascular consequences of participating in war combat. Furthermore, the results are inconclusive. Follow-up mortality rates of male traumatic lower limb amputees of the Israeli army and Vietnam veterans with PTSD had higher mortality rates due to cardiovascular disease, compared with the general population [7,8]. However, recent results from the ARIC study reported that middle-aged veterans after an average of 36 years from combat exposure were not at increased cardiovascular risk compared to controls [9,10]. Furthermore, death rates from circulatory system diseases did not differ between Vietnam veterans and their peers [11] and female Vietnam veterans had significant lower-than-expected deaths from circulatory diseases [12]. On the other hand, results from the studies by Modan, et al [7] and Boscarino [8] are not necessarily conflicting with the studies by Johnson et al [9,10], Boehmer et al [11] and Thomas et al [12], as the former studies looked only at the subset of veterans with physical injury or PTSD, while the latter studies assessed all veterans exposed to combat.

We therefore wanted to pursue the hypothesis that wounded veterans who were alive at the age of 55 are more at long-term risk for coronary death than other veterans. The men in the present study were all born in 1925, and most of them had been involved in war-time activities when they were 17-20 years old. Now, over 60 years later, we have examined whether physical injuries in combat had long-term consequences on coronary mortality in this cohort.

## Methods

### Study population

The Tampere adult population cardiovascular risk study (TAMRISK) is a prospective, longitudinal population based health survey study in Tampere, an urban city in southern Finland with a population of 210 000. All men who were 55 years old in 1980 and residing in Tampere were invited to participate by letters (n = 843). Baseline clinical examinations took place during the calendar year of 1980. The final cohort included 667 men (79% of those invited), who were all Caucasian. The follow-up time was 28 years, from 1980 to 2008. The Ethics Committees of the Tampere University Hospital and the City of Tampere approved the study.

From 1939 to 1945 Finnish troops fought three wars. In November 1939 Winter War broke out with the Soviet Union and lasted for 105 days until March 1940. The men who participated in the Winter War were mainly born in 1910-1921. This was followed by Continuation War from June 1941 to September 1944. Subsequently, until April 1945 there was the Lapland War to expel German troops from Finland. Men who were born in 1919-1921 joined the older age group in the Continuation War. As the war continued, also younger age groups were recruited. The last age group was born in 1925. These men participated, and also fought, when they were 17-20 years old.

Of the 667 men, 527 (79%) reported service in at least one of the wars, 68 (10%) had served outside the front, and 72 (11%) were not in military service. The mean length of service at the front was 1.6 years (range 2.7 years) for those wounded or injured and 1.5 years (range 4.9 years) for those not wounded.

During 28 years of follow-up, there were altogether 412 deaths of all causes, of which 140 from CHD. The men were divided into two groups based on wartime incidents. There were 102 men who had been wounded or injured in action. The remaining 565 subjects who did not report physical injuries served as the comparison group.

### Baseline measurements

The basic evaluation in 1980 included an interview by a trained nurse. The interview was done using a structured questionnaire including questions of vocation, possible disability pension and education. The degree of education at the time of the basic evaluation had been recorded in 1980 on a four-point scale: primary school (n = 440), secondary or vocational school (n = 138), college (n = 54), or university (n = 35). Information on current and previous diseases was based on self-report of diagnosis by a physician, including history of MI and diabetes. The questionnaire also assessed symptoms and ailments experienced within the past six months. These included questions of health in general and mental health, such as anxiety, depression and insomnia. Physical war injury was based on self-report at this baseline examination. Questions of health-related behaviour included current and past smoking. The frequency of physical exercise comprised both leisure and commute related activity. Physical examination included a single blood pressure (BP) measurement (mm of mercury) using a calibrated mercury sphygmomanometer. Serum total cholesterol (mmol/l) was determined from a serum sample by enzymatic techniques. Height (cm) and weight (kg) were measured.

### Outcomes

For the 667 men with complete information, ICD8, ICD9 and ICD10 codes were used to group cause specific mortality into CHD (ICD8 and ICD9: 410-414; ICD10: I20-I25). Vital status was ascertained on the basis of social security number and the cause of death from death certificates. This information was collected up to 2008 from the national statistics centre (Statistics Finland).

### Statistical analyses

Statistical analyses were made using SPSS for Windows program. Dichotomous variables (history of MI, diabetes, smoking) were compared with chi-squared test, continuous variables (blood pressure, total serum cholesterol and body mass index (BMI)) were analyzed with t-test or Mann Whitney U test. Kaplan-Meier survival analysis and Cox proportional hazards regression analysis were used to assess the risk of CHD death. Person-years were calculated from the subjects' examination date and they were censored at the date of death. Those who did not die were censored for the purpose of follow-up time calculation at 25 March 2008.

## Results

Background characteristics of those wounded or injured (n = 102) and for those not wounded (n = 565) are shown in Table 1. Men who had been wounded or injured in action were taller and had slightly higher BMI, had more self-reported depression experienced during the last six months, and were more on disability pension, as compared with the comparison group at the age of 55.

**Table 1:** Background characteristics of men from the TAMRISK study stratified by whether or not they were wounded or injured in World War II

	Comparison group Mean (SD)	Wounded or injured Mean (SD)	p*
N	565	102	
Height (cm)	173.7 (6.1)	175.3 (4.9)	P = 0.003
BMI (kg/m^2^), mean (SD)	26.2 (3.5)	27.0 (3.8)	P = 0.04
Diastolic BP (mm Hg), mean (SD)	91 (9)	92 (10)	NS
Systolic BP (mm Hg), mean (SD)	144 (17)	144 (19)	NS
History of MI % (yes vs. no)	7.2	11.8	NS

Diabetes % (yes vs. no)	4.6	8.0	NS

Educational level (on a scale of 1 to 4)	1.54 (0.87)	1.48 (0.74)	NS

Anxiety within past six months % (yes vs. no)	13.1	15.8	NS

Depression within past six months % (yes vs. no)	12.7	20.8	P = 0.04

Insomnia within past six months % (yes vs. no)	21.1	24.5	NS

Exercise at least every other day % (yes vs. no)	80	78	NS

Health in general % (good/average/poor)	17/65/18	8/70/22	NS

On disability pension % (yes vs. no)	16.9	29.7	P = 0.01

Smoking % (yes vs. no)	38.2	40.0	NS

Ever smoked % (yes vs. no)	82.7	92.2	NS

Serum cholesterol (mmol/l), mean (SD)	6.1 (1.1)	6.0 (1.1)	NS

The men were examined at baseline in 1980 at 55 years of age. *Chi-squared test for dichotomous variables, t-test or Mann Whitney U test for continuous variables. MI, myocardial infarction; BP, blood pressure; BMI, body mass index.

**Figure 1:**
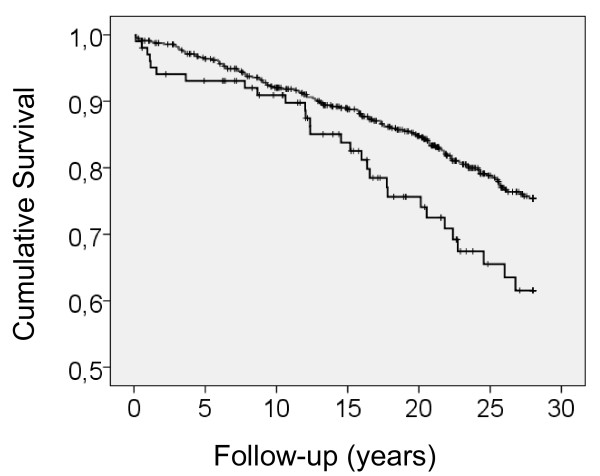
**Kaplan-Meier survival analysis of the comparison group (upper curve) and men wounded or injured in action (lower curve): The men were examined at baseline in 1980 at 55 years of age**.

In Cox proportional hazards analysis, wounded or injured men had a 1.7-fold (95% CI, 1.1-2.5; p = 0.01) increased risk of CHD death. The Kaplan-Meier survival curve illustrates the better survival of the comparison group compared with those who had been wounded or injured in action (Figure 1; p = 0.01). There were significant losses in the group of wounded men in the first 1-2 years of the study followed by a recovery towards years 5-10.

Since the wounded men were significantly taller and heavier and more depressed than their non-wounded peers, we also adjusted for BMI and depression in Cox proportional hazards analysis. After this adjustment, wounded or injured men still had a 1.7-fold (95% CI 1.1-2.5; p = 0.02) increased risk, while BMI only slightly increased the risk for CHD death (RR 1.1; 95% CI 1.0-1.1; p = 0.04), and depression had no effect (RR 0.8; 95% CI 0.5-1.2; p = NS). In men who had been wounded or injured in action the crude CHD mortality rate per 10,000 population was 2843, while in the comparison group the corresponding figure was 1961.

The effect of educational level on the results was also examined. When the information on being wounded or injured in action and the degree of education were both included in Cox proportional hazards analysis, wounded or injured men still had a 1.7-fold (95% CI 1.1-2.6; p = 0.01) increased risk of CHD, while the degree of education had no effect on CHD mortality (RR 0.90, 95% CI 0.77-1.01; p = NS).

## Discussion

Men from Tampere, Finland, who had been wounded or injured in action were 1.7 times more likely to die from CHD than men in the comparison group when over 55 years old. There are only a few previous studies touching on the same subject. Despite small sample size, we feel that this study is an important contribution to the literature because most studies to date have looked at the effects of exposure to combat-related PTSD rather than exposure to combat wound or injury. Further, this study assesses associations within a Finnish population, while the majority of other studies have looked at veterans from other countries (e.g., American, Australian and Middle Eastern populations). Our results are in accordance with a study from the Netherlands, which followed from 1992 to 2002 the overall late-life mortality levels of World War II military veterans, men and women, with birth years from 1920 to 1929. War survivors who had been seriously wounded were at a 1.7-fold risk to live shorter lives [1].

It is notable that there were significant losses in the group of wounded men in the first 1-2 years of the study followed by a recovery towards years 5-10, which might suggest bias towards severely ill individuals during the recruiting of cases in 1980. However, this seems unlikely, since the final cohort included 79% of all the 55-year-old men invited. On the other hand, it is highly probable that we have not seen the whole effect, since the present follow-up was started three decades after the traumatic experience. A follow-up of World War II veterans reported that already within 15 years after the war a subject would experience increased physical decline or death [13]. Likewise, 24-year follow-up mortality rates of male traumatic lower limb amputees of the Israeli army were 1.8-fold higher due to cardiovascular disease, compared with the general population [7]. In a 30-year follow-up, psychotraumatic events among Vietnam veterans were associated with postwar cardiovascular mortality with a hazard ratio of 1.7 [8].

Physical injury is particularly susceptible to concerns about whether trauma influences health outcomes because of some direct injury to the relevant health system, or via biologic damage due to ongoing stress resulting from the trauma. It may be that being wounded is a marker of exposure to severe trauma and only affects health when it induces PTSD or other forms of chronic stress that in turn impose physiologic wear and tear, particularly on the cardiovascular system. The wounded men in our study were more depressed than those in the comparison group, supporting this hypothesis. Effects of early life traumatic events on an individual's health have previously been proposed to extend across the life-course. Greater exposure to deaths of military comrades and exposure to war trauma at a younger age were associated with increased signs of physician-diagnosed cardiac, gastrointestinal, and nervous disease and more unique disease ailments across the life of American Civil War veterans [14]. The siege of Leningrad experienced at pre-pubertal age had also long-term effects leading to higher mortality, especially from ischemic heart disease and circulatory disease [15]. War veterans in this study were very young when exposed to war as soldiers. This may imply that many of them were exposed to war also when too young for the army as civilians, which often means bad nutrition in wartime.

The wounded men in our study had higher BMI and were more often on disability pension, as compared with the comparison group. In addition to depression, also these factors could possibly be intermediary variables between trauma/stress and a CHD death.

Socioeconomic status (SES) has been identified as a major risk factor for coronary disease [16]. A frequent measure of SES is education because it does not usually change after young adulthood. The degree of training had no effect on CHD risk in the present study. A confounding factor might be that over half of the men had no more than basic compulsory education.

In the present study, men who had been wounded or injured in action had higher BMI, as measured three decades after the war, compared to the comparison group. Continuous stressful conditions have previously been reported to lead to distinctive abdominal obesity, as observed in competing sailors during five month offshore sailing races [17]. Since early life traumatic experience may alter systems important in physiological stress regulation, a role for these systems has been speculated in explaining similar findings. In fact, former Finnish child evacuees of Word War II had higher prevalences of cardiovascular disease and type 2 diabetes at the age of 60 [18]. Subjects under continuous stress may develop metabolic alterations towards the metabolic syndrome despite regular exercise and a proper diet. This was especially seen in traumatic lower limb amputees of war. Surviving amputees had hyperinsulinemia, increased coagulability, and increased sympathetic and parasympathetic responses. These established CHD risk factors may thus explain the excess mortality due to CHD [7]. In line with these earlier observations, there was a trend towards an increased prevalence of diabetes and myocardial infarction in our subjects with physical war injuries, compared to the comparison group.

Complete information from 79% (667/843) participants and the long-term follow-up enhance the reliability of our findings. Another strength of the study is that there was no confounding by age or sex because the study group was restricted to Finnish men aged 55 years in 1980; however, this strength also poses a challenge to how broadly one can apply the findings. There are also concerns with exposure measurement, since there was minimal information on the type of injury. There may also be survivorship bias, since the present follow-up was started three decades after the traumatic experience.

## Conclusions

Being physically wounded or injured in action predicted higher CHD mortality compared to the comparison group in a follow-up of Finnish war veterans starting three decades and ending six decades after World War II. Our finding supports the concept that the effects of traumatic events at early adulthood may have long-term consequences on an individual's health. Future studies should delve more into what mechanisms are at work in the observed associations.

## Competing interests

The authors declare that they have no competing interests.

## Authors' contributions

TK and STN had substantial contributions to conception and design and interpretation of data and writing the manuscript. TS had substantial contributions to conception and design. JR and AK participated in data collection. All authors read and approved the final manuscript.

## Pre-publication history

The pre-publication history for this paper can be accessed here:

http://www.biomedcentral.com/1471-2458/11/71/prepub
